# The impact of triglyceride glucose-body mass index on all-cause and cardiovascular mortality in elderly patients with diabetes mellitus: evidence from NHANES 2007–2016

**DOI:** 10.1186/s12877-024-04992-5

**Published:** 2024-04-22

**Authors:** Lei Ding, Bingqi Fu, Hongda Zhang, Cong Dai, Aikai Zhang, Fengyuan Yu, Lijie Mi, Wei Hua, Min Tang

**Affiliations:** 1 Department of Cardiology, State Key Laboratory of Cardiovascular Disease, National Center for Cardiovascular Diseases, Cardiovascular Institute, Fuwai Hospital, Chinese Academy of Medical Sciences, and Peking Union Medical College, Beijing, 100037 China; 2 Department of Health Policy and Management, School of Public Health, Peking University Health Science Center, No.167 Beilishi Rd, Xicheng District, Beijing, 100037 China

**Keywords:** Triglyceride glucose-body mass index, Mortality, Elderly population, Diabetes, NHANES

## Abstract

**Background:**

The relationship between triglyceride glucose-body mass index (TyG-BMI) index and mortality in elderly patients with diabetes mellitus (DM) are still unclear. This study aimed to investigate the association between TyG-BMI with all-cause and cardiovascular mortality among elderly DM patients in the United States (US).

**Methods:**

Patients aged over 60 years with DM from the National Health and Nutrition Examination Survey (2007–2016) were included in this study. The study endpoints were all-cause and cardiovascular mortality and the morality data were extracted from the National Death Index (NDI) which records up to December 31, 2019. Multivariate Cox proportional hazards regression model was used to explore the association between TyG-BMI index with mortality. Restricted cubic spline was used to model nonlinear relationships.

**Results:**

A total of 1363 elderly diabetic patients were included, and were categorized into four quartiles. The mean age was 70.0 ± 6.8 years, and 48.6% of them were female. Overall, there were 429 all-cause deaths and 123 cardiovascular deaths were recorded during a median follow-up of 77.3 months. Multivariate Cox regression analyses indicated that compared to the 1st quartile (used as the reference), the 3rd quartile demonstrated a significant association with all-cause mortality (model 2: HR = 0.64, 95% CI 0.46–0.89, *P* = 0.009; model 3: HR = 0.65, 95% CI 0.43–0.96, *P* = 0.030). Additionally, the 4th quartile was significantly associated with cardiovascular mortality (model 2: HR = 1.83, 95% CI 1.01–3.30, *P* = 0.047; model 3: HR = 2.45, 95% CI 1.07–5.57, *P* = 0.033). The restricted cubic spline revealed a U-shaped association between TyG-BMI index with all-cause mortality and a linear association with cardiovascular mortality, after adjustment for possible confounding factors.

**Conclusions:**

A U-shaped association was observed between the TyG-BMI index with all-cause mortality and a linear association was observed between the TyG-BMI index with cardiovascular mortality in elderly patients with DM in the US population.

## Background

With the aging population expanding, it is expected that by 2050, there would be 2.1 billion people over 60 years old [1]. As age increases, pathophysiological changes such as rise in proinflammatory factors, oxidative stress, mitochondrial dysfunction, and DNA damage can contribute to the development of metabolic disorders [2, 3]. Patients over the age of 60 years make up the majority of diabetes mellitus (DM) cases [4], with the numbers expected to increase further [5]. Elderly diabetic patients are more susceptible to a range of health complications, such as frailty, sarcopenia, cognitive impairment, multimorbidity, coronary artery stenosis, heart failure, stroke and death [6–9]. Early identification of the high-risk patients is essential for improving prognosis.

Insulin resistance (IR) is a state of decreased responsiveness to insulin, and is one of the key pathophysiological features of DM. Increasing evidence suggests that IR contributes to the development of cardiovascular disease (CVD) in individuals with DM and is also an indicator of a poorer prognosis. Due to the high cost and inconvenience associated with traditional methods of evaluating IR, the triglyceride-glucose (TyG) index was developed [10]. TyG index is highly related to hyper-insulinemic euglycemic clamp and the homeostatic model assessment of insulin resistance index (HOMA-IR) [11, 12], and its elevation is positively correlated with poor prognosis [13–16]. Body mass index (BMI) is a simple anthropometric measure of nutritional status, and recent findings indicated that the combination of TyG and BMI (TyG-BMI index) increased effectiveness on assessing IR than TyG index alone [17, 18]. However, the relationship between TyG-BMI index and mortality in elderly patients with DM are still unclear. Thus, the study aimed to investigate the association between TyG-BMI with all-cause and cardiovascular mortality among elderly DM patients in the United States (US).

## Methods

### Study population

**Fig. 1:**
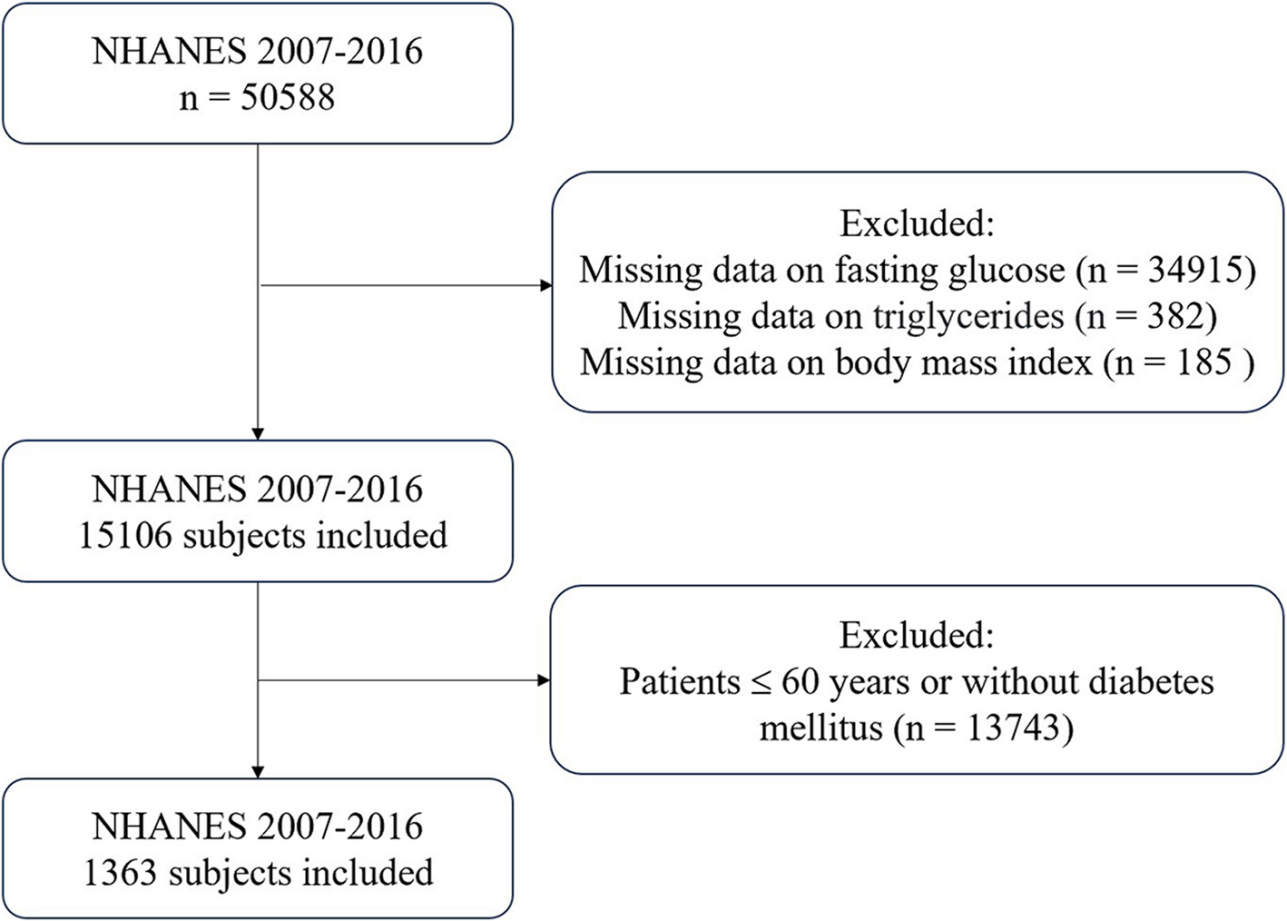
Flowchart of the sample selection from NHANES 2007–2016

Data of this study were collected from the National Health and Nutrition Examination Survey (NHANES), a program managed by the Centers for Disease Control and Prevention (CDC) and the National Centers for Health Statistics (NCHS) in the United States (US). This crucial research program that mainly aims to evaluate the health and nutritional status of Americans and follows the STROBE guidelines for reporting observational studies. The protocols of NHANES have been approved by the Research Ethics Review Board of NCHS and the informed written consent were obtained from all of the participants involved in the study. We extracted the datasets from the NHANES website covering five survey cycles between 2007 and 2016 (https://www.cdc.gov/nchs/nhanes/index.htm). The anonymized NHANES database employs unique identifiers (SEQN) for matching participant data with NDI death records, adhering to CDC's established data linkage protocols. The data generated and analyzed in the study included demographic information, examination data, laboratory data and questionnaire data. Elderly population was defined as older than 60 years old. A total of 50,588 participants were screened in the NHANES cohort between 2007–2016. After excluding those with missing data on fasting plasma glucose (*n* = 34,915), triglycerides (*n* = 382) and BMI (*n* = 185) and those younger than 60 years old or without T2DM (*n* = 13,743), we included 1363 eligible elderly patients with DM in the final analysis (Fig. 1).

### Diagnosis of diabetes

According to the ADA’s diabetes diagnostic criteria, DM was defined as having any of the following: (a) hemoglobin A1C concentration ≥ 6.5% or a fasting plasma glucose level ≥ 126 mg/dL [2]; (b) for those who responded ‘yes’ to the question: ‘Doctor told you have diabetes?’ or ‘Taking insulin now?’.

### Assessment of covariates

Data of demographic, examination, laboratory and questionnaire were collected. BMI was calculated as weight in kilograms divided by height in meters squared. Race/ethnicity was categorized as Mexican American, non-Hispanic white, non-Hispanic black and other race. Alcohol intake was shown as non-drinker, 1 to < 5 drinks/month, 5 to < 10 drinks/month, or 10 + drinks/month. Smoking status was classified as never smoker, former smoker and current smoker. Hypertension was determined by a diagnosed hypertension history by physician, or non-same-day randomized records of 3 times systolic blood pressure ≥ 140 mmHg or diastolic blood pressure ≥ 90 mmHg from baseline interview to 1-year follow-up. Coronary heart disease (CHD) was diagnosed as self-reported CHD, angina pectoris or myocardial infarction. Heart failure was determined by a response of ‘yes’ to the question “Ever told you had heart failure?”. Cancer was defined as self-reported cancer or malignancy. Stroke was determined by a response of ‘yes’ to the question “Ever told you had a stroke?” and chronic obstructive pulmonary disease (COPD) was determined by a response of ‘yes’ to the question “Ever told you had COPD?”. Laboratory data such as fasting plasma glucose, glycosylated hemoglobin type A1c (HbA1c), triglycerides, total cholesterol, low-density lipoprotein cholesterol (LDL-C), and high-density lipoprotein cholesterol (HDL-C) were exported from the NHANES website.

### Assessment of TyG-BMI index

TyG-BMI index = BMI × TyG, where BMI = weight/height^2^, and the TyG index = Ln [fasting triglycerides (mg/dl) × fasting plasma glucose (mg/dl)/2) [1]. The triglycerides and fasting plasma glucose were measured through enzymatic assays on Roche Modular P and Roche Cobas 6000 chemistry analyzers, respectively. All patients were classified into four groups (Q1, Q2, Q3, Q4) by the quartiles of TyG-BMI index, and the group of Q1 was used as the reference group.

### Ascertainment of mortality

The mortality data of the current study were extracted from the National Death Index (NDI) death certificate records provided by NCHS, and the linked mortality files were updated to December 31, 2019. The study endpoints were all-cause mortality and cardiovascular mortality. The International Statistical Classification of Diseases, 10th Revision (ICD-10) was used to determine the reason of deaths. All-cause mortality was defined as death from any cause, including malignant neoplasms (019–043), chronic lower respiratory diseases (082–086), cardiovascular diseases (054–068), and other causes. During follow-up, death due to heart diseases was defined as cardiovascular mortality. The follow-up time was calculated from the baseline interview to the date of death or December 31, 2019.

### Statistical analysis

R software (Version 4.3.2) was used to perform the statistical analyses and create the tables and figures. As required to analyzed the NHANES data, sample weights, clustering, and stratification were incorporated in all analyses [19]. The baseline characteristics were grouped according to quartiles (Q1-Q4) of the TyG-BMI index. Values are means ± standard deviation (SD) for continuous variables. Unweighted frequency counts and weighted percentages are shown for categorical variables. *P* Value was calculated by weighted linear regression model for continuous variables and weighted chi-square test was performed for categorical variables. The incidence rates of all-cause mortality for each TyG-BMI quartile group were computed during the follow-up. In addition, multivariate Cox proportion hazards regression model was applied to identify the independent predictive value of the TyG-BMI index. The Cox regression model included three models to confounding factors. Model 1 was not adjusted any covariates. Model 2 was adjusted for age, gender, race, and total cholesterol. Model 3 was adjusted for age, gender, race, hypertension, total cholesterol, HDL-C, LDL-C, CHD, heart failure, cancer, stroke, and COPD. For missing values, we used multiple imputation. To explore the association between TyG-BMI index and all-cause mortality, Cox proportional hazards regression models with restricted cubic spline (RCS) analyses with four knots. In the RCS model, we also adjusted for confounding factors: age, gender, race, smoking status, alcohol use, hypertension, HDL-C, LDL-C, CHD, heart failure, cancer, stroke, and COPD. Two-piecewise Cox proportional hazards model on both sides of the inflection point was used to investigate the association between SHR and the risk of mortality. Subgroup analyses were also performed to assess the influence of TyG-BMI index on all-cause mortality in different subgroups stratified by age (60–69 years old, and ≥ 70 years old), gender, and race (Mexican American, non-Hispanic white, non-Hispanic black, and other races). A *P* value < 0.05 was defined as the statistical threshold for all analyses.

## Results

### Baseline characteristics

A total of 1363 elderly patients with diabetes were included in the current study cohort (weighted population, 7,261,878). Table 1 showed the baseline characteristics of study patients stratified by quartiles of the TyG-BMI index. The average age of the including patients was 70.0 ± 6.8 years, and 48.6% patients were female. Among these participants, 544 (39.9%) were non-Hispanic White, 154 (11.3%) were Mexican American, 355 (26.0%) were non-Hispanic Black, and 310 (22.7%) were of other races. The mean TyG-BMI index was 286.3 ± 66.5. As for the comorbidities including CHD, heart failure, stroke, cancer and COPD, there was no significant differences between groups (all *P* values > 0.05). Patients with a higher TyG-BMI index were more likely to be younger compared to patients in the lower TyG-BMI index. Importantly, significant differences were observed between the different quartiles, with patients in the highest quartiles have significantly higher levels of total cholesterol and lower levels of HDL-C.

**Table 1:** Baseline characteristics of study participants

Characteristics	Quartiles of TyG-BMI index				
	**Q1 (≤ 236.92)** ***N* = 357**	**Q2 (236.92—278.19)** ***N* = 376**	**Q3 (278.19—324.10)** ***N* = 327**	**Q4 (> 324.10)** ***N* = 303**	***P***
Age (years)	72.4 ± 6.8	71.1 ± 6.7	69.4 ± 6.6	67.2 ± 5.9	< 0.001
Female, n (%)	153 (45.9)	157 (44.6)	171 (51.4)	164 (52.4)	0.424
Height, cm	165.1 ± 9.8	164.9 ± 9.8	165.0 ± 10.3	165.7 ± 10.3	0.770
Weight, kg	66.3 ± 11.0	78.1 ± 11.0	88.8 ± 12.9	84.5 ± 20.7	< 0.001
BMI, kg/m^2^	24.2 ± 2.4	28.6 ± 2.0	32.5 ± 2.3	39.6 ± 5.3	< 0.001
Race/ethnicity, n (%)					< 0.001
Mexican American	31 (8.7)	42 (11.2)	41 (12.5)	40 (13.2)	
Non-Hispanic White	138 (38.7)	146 (38.8)	133 (40.7)	127 (41.9)	
Non-Hispanic Black	79 (22.1)	94 (25.0)	92 (28.1)	90 (29.7)	
Other races	109 (30.5)	94 (25.0)	61 (18.7)	46 (15.2)	
Alcohol use, n (%)					0.165
Non-drinker	119 (36.9)	133 (37.3)	124 (36.8)	115 (31.9)	
1–5 drinks/month	158 (44.6)	171 (50.6)	149 (49.2)	151 (58.1)	
5–10 drinks/month	8 (2.2)	14 (3.7)	6 (1.4)	5 (1.1)	
≥ 10 drinks/month	40 (16.2)	28 (8.4)	25 (12.5)	20 (9.0)	
Smoking status, n (%)					0.213
Never	189 (55.5)	182 (48.8)	157 (47.5)	143 (48.8)	
Former	117 (34.8)	146 (41.5)	148 (48.9)	129 (40.7)	
Current	51 (9.8)	48 (9.8)	22 (3.7)	31 (10.5)	
Hypertension, n (%)	286 (79.3)	302 (79.5)	277 (84.8)	271 (88.9)	0.057
CHD, n (%)	53 (14.8)	69 (18.4)	66 (20.2)	65 (21.5)	0.138
Heart failure, n (%)	33 (9.2)	44 (11.7)	39 (11.9)	48 (15.8)	0.078
Stroke, n (%)	30 (8.4)	44 (11.7)	28 (8.6)	39 (12.9)	0.145
COPD, n (%)	12 (3.4)	14 (3.7)	7 (2.1)	44 (3.2)	0.635
Cancer, n (%)	61 (17.1)	78 (20.7)	67 (20.5)	73 (24.1)	0.174
TG (mmol/L)	1.12 ± 0.6	1.5 ± 0.8	1.8 ± 1.1	2.2 ± 2.0	< 0.001
FPG (mmol/L)	7.7 ± 2.8	8.0 ± 2.8	8.5 ± 2.8	9.2 ± 3.7	< 0.001
TC (mmol/L)	4.4 ± 1.1	4.4 ± 1.0	4.6 ± 1.1	4.7 ± 1.1	0.028
LDL-C (mmol/L)	2.4 ± 0.9	2.4 ± 0.9	2.6 ± 0.9	2.5 ± 0.9	0.369
HDL-C (mmol/L)	1.5 ± 0.7	1.3 ± 0.3	1.2 ± 0.3	1.2 ± 0.3	0.003
Glucose lowering agents					
Insulin	46 (12.9)	63 (16.8)	63 (19.3)	76 (25.1)	< 0.001
Metformin	138 (38.7)	151 (40.2)	145 (44.3)	121 (39.9)	0.469
Acarbose	2 (0.6)	3 (0.8)	1 (0.3)	1 (0.3)	0.780
Sulfonylurea	92 (25.8)	88 (23.4)	100 (30.6)	104 (34.3)	0.008
DPP4 inhibitors	28 (7.8)	20 (5.3)	28 (8.6)	18 (5.9)	0.284
SGLT2 inhibitors	0 (0)	1 (0.3)	1 (0.3)	2 (0.7)	0.484

BMI body mass index, *CHD* coronary heart disease, *COPD* chronic obstructive pulmonary disease, *DPP4* Dipeptidyl Peptidase 4, *FPG* fasting plasma glucose, *TC* total cholesterol, *TG* triglycerides, *LDL-C* low-density lipoprotein cholesterol, *HDL-C* high-density lipoprotein cholesterol, *SGLT2* Sodium-glucose cotransporter-2, *TyG* triglyceride glucose

*P* values are derived using either Student’s t-test or chi-square test

### Multivariate Cox regression analysis for all-cause and cardiovascular mortality

During a mean follow-up period of 77.3 months, 429 all-cause deaths occurred, with an all-cause mortality rate of 402/100000 person-years; 123 cardiovascular deaths occurred, with a cardiovascular mortality rate of 117/100000 person-years. As shown in Table 2, we tried three Cox regression models to investigate the relationship between TyG-BMI index and all-cause mortality. Before adjusted covariates, the hazard ratios (HRs) and confidence intervals (CIs) from the first quartile to the fourth quartile were 1.00 (reference), 0.75 (0.56, 0.99), 0.52 (0.38–0.71), and 0.56 (0.40–0.78), respectively. In model 2, we adjusted for age, gender, race and total cholesterol and the HRs and 95% CIs were 1.00 (reference), 0.85 (0.64–1.15), 0.64 (0.46–0.89), and 0.92 (0.62–1.35), respectively, for all-cause mortality. While in model 3, after adjusting age, gender, race, hypertension, total cholesterol, HDL-C, LDL-C, CHD, heart failure, cancer, stroke, and COPD, the HRs and 95% CIs were 1.00 (reference), 0.83 (0.61–1.13), 0.65 (0.43–0.96), and 0.87 (0.56–1.36), respectively. The multivariate Cox regression models for cardiovascular mortality was shown in Table 3. After adjusting age, gender, race and total cholesterol in model 2, the HRs and 95% CIs from the highest quartile to the lowest quartile were 1.83 (1.01–3.30), 0.73 (0.33–1.61), 1.09 (0.62–1.91), and 1.00 (reference), respectively. In addition, in model 3, we further adjusted hypertension, HDL-C, LDL-C, CHD, heart failure, cancer, stroke, and COPD beyond the model 2. The HRs and 95% CIs were 2.45 (1.07–5.57), 0.99 (0.42–2.32), 1.24 (0.64–2.43), and 1.00 (reference), respectively.

**Table 2:** Association between TYG/BMI index and all-cause mortality

		**Model 1(HR 95%CI)**		**Model 2 (HR 95%CI)**		**Model 3 (HR 95%CI)**	
TyG-BMI index	**Number of deaths**	**HR (95%CI)**	**P**	**HR (95%CI)**	**P**	**HR (95%CI)**	**P**
1st quantile	135	Reference		Reference		Reference	
2nd quantile	129	0.75 (0.56–0.99)	0.043	0.85 (0.64–1.15)	0.294	0.83 (0.61–1.13)	0.239
3rd quantile	78	0.52 (0.38–0.71)	< 0.001	0.64 (0.46–0.89)	0.009	0.65 (0.43–0.96)	0.030
4th quantile	87	0.56 (0.40–0.78)	< 0.001	0.92 (0.62–1.35)	0.653	0.87 (0.56–1.36)	0.544
*P* for trends			< 0.001		0.346		0.350

Model 1: no covariates were adjusted; Model 2: Adjusted for age, gender, race, and total cholesterol; Model 3: Adjusted for age, gender, race, hypertension, total cholesterol, HDL-C, LDL-C, CHD, heart failure, stroke, cancer and COPD

**Table 3:** Association between TYG/BMI index and cardiovascular mortality

		**Model 1(HR 95%CI)**		**Model 2 (HR 95%CI)**		**Model 3 (HR 95%CI)**	
TyG-BMI index	**Number of deaths**	**HR (95%CI)**	**P**	**HR (95%CI)**	**P**	**HR (95%CI)**	**P**
1st quantile	33	Reference		Reference		Reference	
2nd quantile	37	0.94 (0.54–1.67)	0.849	1.09 (0.62–1.91)	0.760	1.24 (0.64–2.43)	0.521
3rd quantile	24	0.59 (0.28–1.26)	0.171	0.73 (0.33–1.61)	0.442	0.99 (0.42–2.32)	0.597
4th quantile	29	0.99 (0.54–1.84)	0.989	1.83 (1.01–3.30)	0.047	2.45(1.07–5.57)	0.033
*P* for trends			0.612		0.131		0.047

Model 1: no covariates were adjusted; Model 2: Adjusted for age, gender, race, and total cholesterol; Model 3: Adjusted for age, gender, race, hypertension, total cholesterol, HDL-C, LDL-C, CHD, heart failure, stroke, cancer and COPD

### Relationships between TyG-BMI index and mortality

**Fig. 2:**
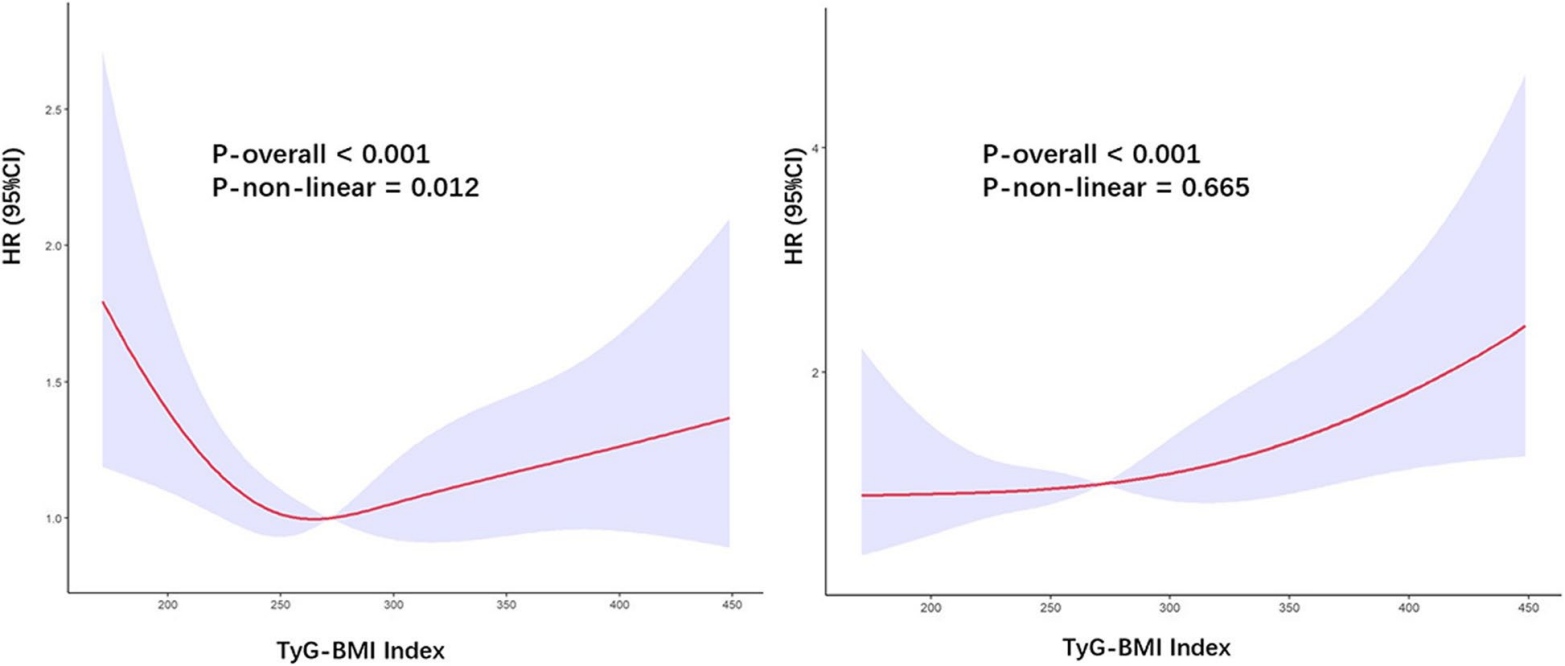
Association between TyG-BMI index and all-cause (**A**) and cardiovascular mortality (**B**) in elderly patients with DM

We used a Cox proportional hazards regression models with RCS to model the relationship between the TyG-BMI index and the risk of mortality in elderly patients with DM. We chose to use four knots, placed at the 25th, 50th, 75th, and 100th percentiles of the distribution of the TyG-BMI index in our study population, to allow for flexible modeling of the non-linear relationship between the TyG-BMI index and mortality. Figure 2A shows the estimated spline function for the relationship between the TyG-BMI index and the risk of all-cause mortality in elderly patients with DM, based on our RCS model. The results indicated a “U-shaped” association after adjusting for age, gender, race, smoking status, alcohol use, hypertension, HDL-C, LDL-C, CHD, heart failure, cancer, stroke, and COPD. *P* value for nonlinearity was < 0.05, when TyG-BMI index was < 264.18, the HR was decreased as the TyG-BMI index increased, while it increased when the TyG-BMI index was > 264.18. The value of TyG-BMI index corresponding to the lowest risk of all-cause mortality on multivariate-adjusted RCS analyses was 264.18 for the population (Fig. 2). Figure 2B shows the estimated spline function for the relationship between the TyG-BMI index and the risk of cardiovascular mortality in elderly patients with DM, based on our RCS model. This RCS model also adjusted the same variables as all-cause mortality. The curve showed an increasing trend in the risk of cardiovascular mortality as the TyG-BMI index increased, and there was a linear relationship with cardiovascular mortality (*P* for non-linear = 0.665). When TyG-BMI index was > 264.18, the HR was increased sharply and TyG-BMI index was served as a risk factor.

### Subgroup analysis

The survival advantages of a higher TyG-BMI index (≥ 264.18 for all-cause mortality) compared to a lower TyG-BMI index (< 264.18 for all-cause mortality) among elderly patients with DM was consistent across various subgroups based on age, gender and race as depicted in Table 4. There was no significant interaction between the TyG-BMI and stratified variables.

**Table 4:** Subgroup analyses of the association between TYG-BMI index and all-cause mortality

TyG-BMI index	< 264.18		≥ 264.18		*P* interaction
	**HR (95%CI)**	**P**	**HR (95%CI)**	**P**	
**Age, years**					0.180
60–69	Reference		0.99 (0.57, 1.73)	0.983	
≥ 70	Reference		0.63 (0.49, 0.81)	< 0.001	
**Gender**					0.198
Female	Reference		0.53 (0.39, 0.73)	< 0.001	
Male	Reference		0.75 (0.52, 1.08)	0.118	
**Race/ethnicity**					0.145
Mexican American	Reference		1.96 (0.65, 5.97)	0.235	
Non-Hispanic White	Reference		0.59 (0.44, 0.78)	< 0.001	
Non-Hispanic Black	Reference		0.61 (0.41, 0.92)	0.018	
Other races	Reference		0.73 (0.37, 1.44)	0.364	

## Discussion

To the best of our knowledge, this is the first study to evaluate the association between TyG-BMI with all-cause and cardiovascular mortality among elderly diabetes patients. The major findings of the current study were as follows: (1) a significant correlation between the TyG-BMI index and all-cause and cardiovascular mortality in old diabetic patients, even after adjusting for potential confounding variables; (2) a U-shaped relationship was observed between baseline TyG-BMI index and all-cause mortality; (3) a linear relationship was observed between baseline TyG-BMI index and cardiovascular mortality.

IR is well acknowledged for its role in pathogenesis of type 2 DM, dyslipidemias, and obesity in general population [20, 21]. In addition, IR is linked to the development of CVD, and serves as a predictor for cardiovascular outcomes and all-cause mortality [22–24]. TyG-BMI index, which integrates TG, fasting plasma glucose and BMI, serves as a simple and feasible assessment tool for IR [25]. Many studies supported the close association between TyG-BMI index and visceral adiposity [26], type 2 DM [27], and metabolic syndrome [28]. The clinical value of TyG-BMI index on predicting occurrence of hypertension [29], nonalcoholic fatty liver disease [30], DM [31], and severe coronary artery disease [32] have been demonstrated in previous studies. Apart from the substantial correlation with various illness, TyG-BMI index was also useful for predicting clinical outcomes. In patients with heart failure, lower TyG-BMI index was associated with higher risk of one-year mortality [33]. In elderly population undergoing percutaneous coronary intervention, lower TyG-BMI index was correlated with higher risk for major adverse cardiac and cerebrovascular events [34]. These evidences implied that TyG-BMI index could become a valuable predictor for prognosis.

In our study, TyG-BMI index revealed similar predictive value for all-cause mortality in old diabetic patients; specifically, a U-shaped relationship between baseline TyG-BMI index and all-cause mortality was revealed. The U-shaped association between IR indicator and poor prognosis has been established in previous studies utilizing NHANES data. Zhang et al. observed a U-shaped association between TyG index with all-cause mortality in CVD patients with DM or pre-DM [35]. Sun et al. found a similar U-shaped association between TyG index and all-cause mortality in elderly US population [36]. The results were consistent in Li et al.’s study, where a U-shaped association was revealed with all-cause mortality in patients with CVD [37]. The mechanisms underlying this U-shaped association remain uncertain, but they may encompass the following aspects.

To begin with, a high TyG index is indicative of hyperglycemia and high TG level, were associated with adverse outcomes. Persistent hyperglycemia is related to a higher risk of death in nondiabetic patients with intracerebral hemorrhage [38]. For ischemic stroke patients, hyperglycemia is correlated with early neurological degeneration, 3-month poor outcome, and mortality after thrombolysis [39]. Uncontrolled hyperglycemia in the long run is associated with increased risk of micro- and macrovascular complications and mortality [40, 41]. High TG level, on the other hand, is an important contributor of residual cardiovascular risk. It may play a role on the development of CVD [42]. Marston et al. conducted meta-analysis of randomized controlled trials, and supported that lowering TG was associated lower risk of major vascular events [43]. Both hyperglycemia and high TG level can initiate oxidative stress, intensify inflammatory responses, promote the formation of foam cells, impair endothelial function, stimulate the proliferation of smooth muscle cells, escalate sympathetic nervous system activity, enhance renal sodium retention, raise blood pressure, and consequently increase cardiac load and lead to vascular damage [42, 44, 45].

Furthermore, a low TyG index reflects hypoglycemia and low TG level, and could also be harmful. Firstly, hypoglycemia induces platelet hyperactivity through the elevation of inflammatory and oxidative stress markers [46, 47]. It stimulates the production of adrenaline, a potent counter-regulatory hormone that further enhances platelet activation [48]. As a result, the platelet-related protein responses triggered by hypoglycemia increase the risk of cardiovascular and cerebrovascular events [49, 50]. Secondly, prior researches indicated that low TG level were associated with increased risk for hemorrhagic stroke [51–54], cardiac death in heart failure [55], in-hospital and late major adverse events in patients with ST-segment elevation myocardial infarction treated with primary percutaneous coronary intervention [56], increased incident DM in normoglycemic males with non-alcoholic fatty liver disease [57], and mortality in septic patients [58]. The situation is referred as “TG paradox”. The underlying mechanism is yet unclear, but might be explained by the inability to maintain a stable state of cell membrane and poor nutrition under the circumstance of low TG level [59].

Last but not least, BMI measures the amount of body fat and offers insights into nutritional status, and when compared to TyG index alone, TyG-BMI index is believed to possess an augmented risk assessment capacity, owing to its inclusion of obesity- and malnutrition-related information [27]. The relationship between both overweight or obese individuals and increased mortality risk has been demonstrated in large cohort studies recruiting general population [60]. Obesity is directly linked to the development of dyslipidemia, type 2 DM, and hypertension [61], and is associated with CVD and cardiac death. The primary mechanisms underlying these associations involve the production of inflammatory mediators, reactive oxidative stress, and IR [62]. More recent findings, however, found a U-shaped relationship between BMI and mortality in patients with DM, hypertension, heart failure, and CAD [63, 64]. Malnutrition is a risk factor for unfavorable outcomes in hospitalized patients [65], possibly via increasing circulatory inflammatory mediators, worsen IR and disturbing metabolic states [66]. Particularly, in old population, malnutrition is associated with frailty, cachexia, sarcopenia [67], and a strong predictor for worse clinical outcomes [68].

To sum it up, TG, fasting plasma glucose and BMI are integral components of the TyG-BMI index. Previous studies have demonstrated a U-shaped association of each of these components with clinical outcomes. These findings, in conjunction with our own, highlight the clinical significance of maintaining an optimal TyG-BMI index, as deviations to either extremely high or low levels can result in detrimental health consequences.

Our study had limited data access to diabetic-significant variables such as previous hypoglycemic events, which may significantly influence the outcomes in our study population. Future research should integrate a more comprehensive array of variables that have demonstrated significance in influencing outcomes among diabetic populations. Second of all, despite adjusting for potential confounding factors in the multivariate model and subgroup analyses, the inherent nature of observational studies and the restricted sample size for each subgroup necessitate careful interpretation of results due to the possibility of residual confounders. Finally, the lack of significance in the univariate cox regression for TyG-BMI and cardiovascular death and the absence of a similar U-shaped association could be attributed to the significantly younger age in the highest TyG-BMI quartile, which was not adjusted for in the univariate analysis but was in multivariate models 2 and 3. As our observational retrospective cohort study has a low incidence of cardiovascular death, the relationship between TyG-BMI and cardiovascular outcomes should be further investigated in randomized controlled trials with larger cohorts.

## Conclusions

In the current study, the TyG-BMI index is a valuable index for predicting the risk of all-cause and cardiovascular mortality in elderly patients withDM, the association between the TyG-BMI index and all-cause mortality is U shaped. While the association between the TyG-BMI index and cardiovascular mortality is linear. The TyG-BMI index can serve as a convenient tool for identifying IR, may be beneficial in assessing the risk and predicting the prognosis of elderly diabetes patients.

## Data Availability

The datasets presented in this article are not readily available because research data is confidential. Data sharing requests are required to meet the policies of the hospital and the funder. Requests to access the datasets should be directed to doctortangmin@yeah.net.

## References

[1] World Health Organization. Ageing. Available at: https://www.who.int/health-topics/ageing#tab=tab_1. Accessed 07 Apr. 2024

[2] Bechtold M, Palmer J, Valtos J, Iasiello C, Sowers J (2006). Metabolic syndrome in the elderly. Curr Diab Rep.

[3] Longo M, Bellastella G, Maiorino MI, Meier JJ, Esposito K, Giugliano D (2019). Diabetes and aging: from treatment goals to pharmacologic therapy. Front Endocrinol (Lausanne).

[4] Decode Study Group (2003). Age- and sex-specific prevalences of diabetes and impaired glucose regulation in 13 European cohorts. Diabetes Care.

[5] Cho NH, Shaw JE, Karuranga S, Huang Y, da Rocha Fernandes JD, Ohlrogge AW, Malanda B (2018). IDF Diabetes Atlas: Global estimates of diabetes prevalence for 2017 and projections for 2045. Diabetes Res Clin Pract.

[6] Bellary S, Kyrou I, Brown JE, Bailey CJ (2021). Type 2 diabetes mellitus in older adults: clinical considerations and management. Nat Rev Endocrinol.

[7] Inciardi RM, Claggett B, Gupta DK, Cheng S, Liu J, Echouffo Tcheugui JB, Ndumele C, Matsushita K, Selvin E, Solomon SD (2022). Cardiac structure and function and diabetes-related risk of death or heart failure in older adults. J Am Heart Assoc.

[8] Obisesan OH, Orimoloye OA, Wang FM, Dardari ZA, Selvin E, Boakye E, Osei AD, Honda Y, Dzaye O, Pankow J (2023). Coronary artery calcium scores in older adults with diabetes and their association with diabetes-specific risk enhancers (from the atherosclerosis risk in communities study). Am J Cardiol.

[9] Virani SS, Alonso A, Benjamin EJ, Bittencourt MS, Callaway CW, Carson AP, Chamberlain AM, Chang AR, Cheng S, Delling FN (2020). Heart disease and stroke statistics-2020 update: a report from the American Heart Association. Circulation.

[10] Simental-Mendia LE, Rodriguez-Moran M, Guerrero-Romero F (2008). The product of fasting glucose and triglycerides as surrogate for identifying insulin resistance in apparently healthy subjects. Metab Syndr Relat Disord.

[11] Guerrero-Romero F, Simental-Mendia LE, Gonzalez-Ortiz M, Martinez-Abundis E, Ramos-Zavala MG, Hernandez-Gonzalez SO, Jacques-Camarena O, Rodriguez-Moran M (2010). The product of triglycerides and glucose, a simple measure of insulin sensitivity. Comparison with the euglycemic-hyperinsulinemic clamp. J Clin Endocrinol Metab.

[12] Vasques AC, Novaes FS, de Oliveira MS, Souza JR, Yamanaka A, Pareja JC, Tambascia MA, Saad MJ, Geloneze B (2011). TyG index performs better than HOMA in a Brazilian population: a hyperglycemic clamp validated study. Diabetes Res Clin Pract.

[13] Park K, Ahn CW, Lee SB, Kang S, Nam JS, Lee BK, Kim JH, Park JS (2019). Elevated TyG index predicts progression of coronary artery calcification. Diabetes Care.

[14] Won KB, Park EJ, Han D, Lee JH, Choi SY, Chun EJ, Park SH, Han HW, Sung J, Jung HO (2020). Triglyceride glucose index is an independent predictor for the progression of coronary artery calcification in the absence of heavy coronary artery calcification at baseline. Cardiovasc Diabetol.

[15] Li H, Zuo Y, Qian F, Chen S, Tian X, Wang P, Li X, Guo X, Wu S, Wang A (2022). Triglyceride-glucose index variability and incident cardiovascular disease: a prospective cohort study. Cardiovasc Diabetol.

[16] Wu Z, Liu L, Wang W, Cui H, Zhang Y, Xu J, Zhang W, Zheng T, Yang J (2022). Triglyceride-glucose index in the prediction of adverse cardiovascular events in patients with premature coronary artery disease: a retrospective cohort study. Cardiovasc Diabetol.

[17] Bala C, Gheorghe-Fronea O, Pop D, Pop C, Caloian B, Comsa H, Bozan C, Matei C, Dorobantu M (2019). The association between six surrogate insulin resistance indexes and hypertension: a population-based study. Metab Syndr Relat Disord.

[18] Ramirez-Velez R, Perez-Sousa MA, Gonzalez-Ruiz K, Cano-Gutierrez CA, Schmidt-RioValle J, Correa-Rodriguez M, Izquierdo M, Romero-Garcia JA, Campos-Rodriguez AY, Triana-Reina HR (2019). Obesity- and lipid-related parameters in the identification of older adults with a high risk of prediabetes according to the American diabetes association: an analysis of the 2015 health, well-being, and aging study. Nutrients.

[19] NHANES Survey Methods and Analytic Guidelines. https://wwwn.cdc.gov/nchs/nhanes/analyticguidelines.aspx. Accessed 9 Dec 2023

[20] Rohm TV, Meier DT, Olefsky JM, Donath MY (2022). Inflammation in obesity, diabetes, and related disorders. Immunity.

[21] Santoro A, Kahn BB (2023). Adipocyte regulation of insulin sensitivity and the risk of type 2 diabetes. N Engl J Med.

[22] Hill MA, Yang Y, Zhang L, Sun Z, Jia G, Parrish AR, Sowers JR (2021). Insulin resistance, cardiovascular stiffening and cardiovascular disease. Metabolism.

[23] Di Pino A, DeFronzo RA (2019). Insulin resistance and atherosclerosis: implications for insulin-sensitizing agents. Endocr Rev.

[24] Pan K, Nelson RA, Wactawski-Wende J, Lee DJ, Manson JE, Aragaki AK, Mortimer JE, Phillips LS, Rohan T, Ho GYF (2020). Insulin resistance and cancer-specific and all-cause mortality in postmenopausal women: the women's health initiative. J Natl Cancer Inst.

[25] Lim J, Kim J, Koo SH, Kwon GC (2019). Comparison of triglyceride glucose index, and related parameters to predict insulin resistance in Korean adults: an analysis of the 2007–2010 Korean national health and nutrition examination survey. Plos One.

[26] Er LK, Wu S, Chou HH, Hsu LA, Teng MS, Sun YC, Ko YL (2016). Triglyceride glucose-body mass index is a simple and clinically useful surrogate marker for insulin resistance in nondiabetic individuals. Plos One.

[27] Kuang M, Yang R, Huang X, Wang C, Sheng G, Xie G, Zou Y (2023). Assessing temporal differences in the predictive power of baseline TyG-related parameters for future diabetes: an analysis using time-dependent receiver operating characteristics. J Transl Med.

[28] Gui J, Li Y, Liu H, Guo LL, Li J, Lei Y, Li X, Sun L, Yang L, Yuan T (2023). Obesity- and lipid-related indices as a predictor of obesity metabolic syndrome in a national cohort study. Front Public Health.

[29] Cheng W, Kong F, Chen S (2022). Comparison of the predictive value of four insulin resistance surrogates for the prevalence of hypertension: a population-based study. Diabetol Metab Syndr.

[30] Li H, Shi Z, Chen X, Wang J, Ding J, Geng S, Sheng X, Shi S (2023). Relationship between six insulin resistance surrogates and nonalcoholic fatty liver disease among older adults: a cross-sectional study. Diabetes Metab Syndr Obes.

[31] Han Y, Hu H, Li Q, Deng Z, Liu D (2023). Triglyceride glucose-body mass index and the risk of progression to diabetes from prediabetes: A 5-year cohort study in Chinese adults. Front Public Health.

[32] Zhang Y, Wang R, Fu X, Song H (2022). Non-insulin-based insulin resistance indexes in predicting severity for coronary artery disease. Diabetol Metab Syndr.

[33] Dou J, Guo C, Wang Y, Peng Z, Wu R, Li Q, Zhao H, Song S, Sun X, Wei J (2023). Association between triglyceride glucose-body mass and one-year all-cause mortality of patients with heart failure: a retrospective study utilizing the MIMIC-IV database. Cardiovasc Diabetol.

[34] Cheng Y, Fang Z, Zhang X, Wen Y, Lu J, He S, Xu B (2023). Association between triglyceride glucose-body mass index and cardiovascular outcomes in patients undergoing percutaneous coronary intervention: a retrospective study. Cardiovasc Diabetol.

[35] Zhang Q, Xiao S, Jiao X, Shen Y (2023). The triglyceride-glucose index is a predictor for cardiovascular and all-cause mortality in CVD patients with diabetes or pre-diabetes: evidence from NHANES 2001–2018. Cardiovasc Diabetol.

[36] Sun M, Guo H, Wang Y, Ma D (2022). Association of triglyceride glucose index with all-cause and cause-specific mortality among middle age and elderly US population. BMC Geriatr.

[37] Li H, Jiang Y, Su X, Meng Z (2023). The triglyceride glucose index was U-shape associated with all-cause mortality in population with cardiovascular diseases. Diabetol Metab Syndr.

[38] Qureshi AI, Huang W, Lobanova I, Chandrasekaran PN, Hanley DF, Hsu CY, Martin RH, Steiner T, Suarez JI, Yamamoto H (2022). Effect of moderate and severe persistent hyperglycemia on outcomes in patients with intracerebral hemorrhage. Stroke.

[39] Cheng Y, Ying A, Lin Y, Yu J, Luo J, Zeng Y, Lin Y (2020). Neutrophil-to-lymphocyte ratio, hyperglycemia, and outcomes in ischemic stroke patients treated with intravenous thrombolysis. Brain Behav.

[40] Husemoen LLN, Morch LS, Christensen PK, Hartvig NV, Feher MD (2021). All-cause and cardiovascular mortality among insulin-naive people with type 2 diabetes treated with insulin detemir or glargine: a cohort study in the UK. Diabetes Ther.

[41] Oh SH, Kim D, Hwang J, Kang JH, Kwon Y, Kwon JW (2023). Association of uncontrolled hypertension or diabetes mellitus with major adverse cardiovascular events and mortality in South Korea: population-based cohort study. JMIR Public Health Surveill.

[42] Sandesara PB, Virani SS, Fazio S, Shapiro MD (2019). The forgotten lipids: triglycerides, remnant cholesterol, and atherosclerotic cardiovascular disease risk. Endocr Rev.

[43] Marston NA, Giugliano RP, Im K, Silverman MG, O'Donoghue ML, Wiviott SD, Ference BA, Sabatine MS (2019). Association between triglyceride lowering and reduction of cardiovascular risk across multiple lipid-lowering therapeutic classes: a systematic review and meta-regression analysis of randomized controlled trials. Circulation.

[44] Gao S, Ma W, Huang S, Lin X, Yu M (2021). Impact of triglyceride-glucose index on long-term cardiovascular outcomes in patients with myocardial infarction with nonobstructive coronary arteries. Nutr Metab Cardiovasc Dis.

[45] da Silva AA, do Carmo JM, Li X, Wang Z, Mouton AJ, Hall JE (2020). Role of hyperinsulinemia and insulin resistance in hypertension: metabolic syndrome revisited. Can J Cardiol.

[46] Kahal H, Halama A, Aburima A, Bhagwat AM, Butler AE, Graumann J, Suhre K, Sathyapalan T, Atkin SL (2020). Effect of induced hypoglycemia on inflammation and oxidative stress in type 2 diabetes and control subjects. Sci Rep.

[47] Halama A, Kahal H, Bhagwat AM, Zierer J, Sathyapalan T, Graumann J, Suhre K, Atkin SL (2019). Metabolic and proteomic signatures of hypoglycaemia in type 2 diabetes. Diabetes Obes Metab.

[48] Yamamoto K, Ito T, Nagasato T, Shinnakasu A, Kurano M, Arimura A, Arimura H, Hashiguchi H, Deguchi T, Maruyama I (2019). Effects of glycemic control and hypoglycemia on Thrombus formation assessed using automated microchip flow chamber system: an exploratory observational study. Thromb J.

[49] Moin ASM, Sathyapalan T, Atkin SL, Butler AE (2022). The severity and duration of Hypoglycemia affect platelet-derived protein responses in Caucasians. Cardiovasc Diabetol.

[50] Klingbeil KD, Koch S, Dave KR (2020). Potential link between post-acute ischemic stroke exposure to hypoglycemia and hemorrhagic transformation. Int J Stroke.

[51] Rist PM, Buring JE, Ridker PM, Kase CS, Kurth T, Rexrode KM (2019). Lipid levels and the risk of hemorrhagic stroke among women. Neurology.

[52] Sturgeon JD, Folsom AR, Longstreth WT, Shahar E, Rosamond WD, Cushman M (2007). Risk factors for intracerebral hemorrhage in a pooled prospective study. Stroke.

[53] Wieberdink RG, Poels MM, Vernooij MW, Koudstaal PJ, Hofman A, van der Lugt A, Breteler MM, Ikram MA (2011). Serum lipid levels and the risk of intracerebral hemorrhage: the Rotterdam study. Arterioscler Thromb Vasc Biol.

[54] Bonaventure A, Kurth T, Pico F, Barberger-Gateau P, Ritchie K, Stapf C, Tzourio C (2010). Triglycerides and risk of hemorrhagic stroke vs. ischemic vascular events: The Three-City study. Atherosclerosis..

[55] Kozdag G, Ertas G, Emre E, Akay Y, Celikyurt U, Sahin T, Gorur G, Karauzum K, Yilmaz I, Ural D (2013). Low serum triglyceride levels as predictors of cardiac death in heart failure patients. Tex Heart Inst J.

[56] Cheng YT, Liu TJ, Lai HC, Lee WL, Ho HY, Su CS, Liu CN, Wang KY (2014). Lower serum triglyceride level is a risk factor for in-hospital and late major adverse events in patients with ST-segment elevation myocardial infarction treated with primary percutaneous coronary intervention- a cohort study. BMC Cardiovasc Disord.

[57] Xie X, Liao J, Huang C, Li X, Cao Q, Kong L, Okamura T, Hashimoto Y, Obora A, Kojima T (2023). U-shaped association between triglyceride and risk of incident diabetes in normoglycemic males with NAFLD: a population-base cohort study. Int J Med Sci.

[58] Xiao M, Deng H, Mao W, Liu Y, Yang Q, Liu Y, Fan J, Li W, Liu D (2023). U-shaped association between serum triglyceride levels and mortality among septic patients: an analysis based on the MIMIC-IV database. Plos One.

[59] Xia TL, Li YM, Huang FY, Chai H, Huang BT, Li Q, Zhao ZG, Liao YB, Zuo ZL, Peng Y (2019). The triglyceride paradox in the mortality of coronary artery disease. Lipids Health Dis.

[60] Heymsfield SB, Wadden TA (2017). Mechanisms, pathophysiology, and management of obesity. N Engl J Med.

[61] Powell-Wiley TM, Poirier P, Burke LE, Despres JP, Gordon-Larsen P, Lavie CJ, Lear SA, Ndumele CE, Neeland IJ, Sanders P (2021). Obesity and cardiovascular disease: a scientific statement from the American heart association. Circulation.

[62] La Sala L, Pontiroli AE (2020). Prevention of diabetes and cardiovascular disease in obesity. Int J Mol Sci..

[63] Dwivedi AK, Dubey P, Cistola DP, Reddy SY (2020). Association between obesity and cardiovascular outcomes: updated evidence from meta-analysis studies. Curr Cardiol Rep.

[64] Sharma A, Lavie CJ, Borer JS, Vallakati A, Goel S, Lopez-Jimenez F, Arbab-Zadeh A, Mukherjee D, Lazar JM (2015). Meta-analysis of the relation of body mass index to all-cause and cardiovascular mortality and hospitalization in patients with chronic heart failure. Am J Cardiol.

[65] Guenter P, Abdelhadi R, Anthony P, Blackmer A, Malone A, Mirtallo JM, Phillips W, Resnick HE (2021). Malnutrition diagnoses and associated outcomes in hospitalized patients: United States, 2018. Nutr Clin Pract.

[66] Yoo JE, Han K, Jung JH, Hur YI, Kim YH, Kim ES, Son JW, Rhee EJ, Lee WY, Nam GE (2023). Body mass index, waist circumference and cardiovascular diseases in transitional ages (40 and 66 years). J Cachexia Sarcopenia Muscle.

[67] Bullock AF, Greenley SL, McKenzie GAG, Paton LW, Johnson MJ (2020). Relationship between markers of malnutrition and clinical outcomes in older adults with cancer: systematic review, narrative synthesis and meta-analysis. Eur J Clin Nutr.

[68] Bellanti F, Lo Buglio A, Quiete S, Vendemiale G (2022). Malnutrition in hospitalized old patients: screening and diagnosis, clinical outcomes, and management. Nutrients.

